# School District Leader Perspectives on Surveying Middle School Youth About Sexual Violence

**DOI:** 10.1111/josh.13496

**Published:** 2024-08-07

**Authors:** Avanti Adhia, Ruby Lucas, Ann E. Richey, Megan Rogers, Nikki Van Wagner, Laurie Dils, Frederick P. Rivara, Betty Bekemeier

**Affiliations:** a Department of Child, Family, and Population Health Nursing, University of Washington, Seattle, WA; Department of Epidemiology, School of Public Health, University of Washington, Seattle, WA.; b Department of Epidemiology, School of Public Health, University of Washington, Seattle, WA; Department of Health Systems and Population Health, School of Public Health, University of Washington, Seattle, WA; Northwest Center for Public Health Practice, University of Washington, Seattle, WA.; c Department of Epidemiology, School of Public Health, University of Washington, Seattle, WA.; d Department of Health Systems and Population Health, School of Public Health, University of Washington, Seattle, WA; Northwest Center for Public Health Practice, University of Washington, Seattle, WA.; e Sexual Violence Prevention, Office of Superintendent of Public Instruction, Olympia, WA.; f Health and Sexual Health Education, Office of Superintendent of Public Instruction, Olympia, WA.; g Department of Epidemiology, School of Public Health, University of Washington, Seattle, WA; Department of Pediatrics, University of Washington, Seattle, WA.; h Department of Child, Family, and Population Health Nursing, University of Washington, Seattle, WA; Department of Health Systems and Population Health, School of Public Health, University of Washington, Seattle, WA; Northwest Center for Public Health Practice, University of Washington, Seattle, WA.

**Keywords:** sexual violence, adolescents, surveys, middle school, school board members, superintendents

## Abstract

**BACKGROUND:**

Schools are important contexts for preventing sexual violence (SV) among adolescents. Evaluating whether programming is effective requires surveying youth about SV experiences. However, school communities often have concerns about asking students, particularly those in middle school, about these experiences. This study sought to understand the types of concerns that school district leaders have related to surveying middle school students about SV and to identify ways to mitigate these concerns.

**METHODS:**

We conducted semi-structured interviews with superintendents and school board members (n = 19) across Washington State and used inductive thematic analysis.

**RESULTS:**

Concerns regarding surveying students about SV centered around 3 main themes: community norms and misconceptions, parental/caregiver discomfort, and survey language and administration. Concerns were particularly salient for sixth-grade students. Suggestions for mitigating concerns included: providing clear motivation and reframing messaging to community members, involving parents and students in the survey process, and modifying survey language and administration.

**CONCLUSIONS:**

Researchers administering surveys to middle school students on sensitive topics including SV may face pushback and must consider flexible approaches to allow research and evaluation to be conducted.

Adolescence is a period of particularly high risk of sexual violence (SV), including sexual activity without consent (eg, unwanted touching, sexual harassment, sexual assault).^1^ Over half of women who experience unwanted sexual contact first do so before age 18, with 22% first victimized when they are 10 years or younger.^2^ Early adolescence—including middle school (grades 6–8)—is a critical developmental period for precursors of SV and early SV experiences. Among middle school students, SV can include lewd or harassing comments about someone’s body or sexuality, sharing photos without consent, sending unwanted sexual messages, homophobic teasing, and coerced sexual contact.^3–5^ Given that SV is associated with myriad negative health consequences such as depression, substance use, and poor academic performance,^6,7^ prevention of SV is a priority.

School-based programs are effective in preventing adolescent SV and other forms of interpersonal violence, including among middle and high school students.^8–10^ Comprehensive sexual health education can also improve knowledge and skills, supporting healthy relationships and decreasing violence.^11^ Research emphasizes the need for early and scaffolded approaches to prevention.^11^ Moreover, SV prevention aligns with national education standards for health and sexuality, which advise including SV and interpersonal violence.^12,13^ In December 2020, Washington State passed a law mandating comprehensive sexual health education in public schools, including content about interpersonal relationship skills, healthy sexual relationships, consent, bystander training, and age-appropriate information on sexual offenses.^14^

Surveying students is required to understand the burden of SV and to evaluate effectiveness of prevention programming, especially given low rates of formal reporting of SV.^15^ These surveys often include questions related to SV knowledge, attitudes, and victimization and/or perpetration of specific behaviors.^8,9^ School district leaders and families sometimes have concerns about asking students, particularly those in middle school, these types of questions. Previous studies with parents about SV education and surveys identified barriers like denial of or discomfort with the topic, fear about introducing SV to children (eg, loss of innocence), and beliefs in parental responsibility for protection against SV.^16,17^ In addition, school staff face challenges in implementing school-wide SV prevention due to school culture and relatively low priority of SV compared to other school concerns.^18^

Given the importance of SV prevention among youth, strategies to effectively partner with schools and ensure rigorous evaluation of programming are needed. Our study had 2 aims: (1) to understand the types of concerns related to surveying students about SV from the perspective of school district leaders (eg, superintendents and school board members), and (2) to identify ways to mitigate these concerns to allow research and evaluation to be conducted. The current study was part of a larger evaluation of an SV prevention program being developed and piloted in nonurban middle schools in Washington State, which involved data collection from students via surveys.^19^ While schools involve many invested parties (eg, parents, staff), superintendents and school boards often have high-level decision-making authority. More recently, school leadership has been plagued by controversy, such as threats related to COVID-19 public health measures.^20^ Investigating contemporary attitudes about surveying students can inform current and future research-practice efforts to address SV.

## METHODS

### Participants

We interviewed superintendents and school board members associated with public middle schools across Washington State. We used purposive sampling to ensure representation from nonurban school districts, given the focus of the pilot program and because urban and nonurban districts may differ in factors that influence attitudes about surveying students. We divided Washington into 6 quadrants and chose 2 districts in each (large and small). We encountered substantial nonresponse to email invitations to participate and contacted potential participants in waves if individuals did not respond. In total, we contacted school board chairs and district superintendents in 48 districts (out of 295 total districts in the state). The research team sent up to 3 interview requests to each individual. Our final sample represented 13 districts. We received no response from 31 districts, 2 declined participation, and 2 initially responded but were unable to schedule interviews. As we interviewed participants, we used snowball sampling to identify additional participants who would share alternative perspectives. Two participants were recruited this way. We continued recruitment until we reached sufficient information power based on the specificity of our research questions, participants with shared and relevant experiences in school leadership, and dialogue quality centered around focused interview questions on surveying students about SV.^21^ We also monitored geographic representation to ensure we were covering different regions of the state and differing levels of urbanicity of school districts. While we were conducting interviews, we had regular full research team discussions (including the interviewers and notetakers) and began preliminary analyses in parallel. In addition, we found school leaders from diverse districts had similar concerns and recommendations related to our interview questions. As a team, we thus decided to end recruitment after 17 interviews with 19 participants representing 13 districts. By participant request, 2 interviews were conducted with 2 participants from 1 district simultaneously. Some participants were parents themselves with children in school. In addition, some participants were concerned about having their school district or identity revealed, so we do not report identifying information about participants and districts. The study was approved by the University of Washington Institutional Review Board.

### Instrumentation and Procedure

We conducted 60-minute interviews on Zoom between July and November 2022. The interviewer provided a study overview, including a statement of confidentiality, and participants verbally consented to participate. Interview topics included school community (eg, administrators, staff, parents) attitudes and concerns about SV surveys, acceptable language in survey questions, and strategies to alleviate concerns about surveys. Participants were also shown example survey questions for their reactions (see Supporting Information). While our unit of analysis was individual participants, we did not collect demographic data on individuals or provide school district names given their hesitation about identifiability. For context about the student populations that school leaders served, we provide characteristics of included school districts (eg, student demographics, size, student-teacher ratio).^22^

### Data Analysis

Descriptive statistics on district characteristics were calculated using R Studio and Excel. Interviews were recorded, transcribed, and analyzed using ATLAS.ti version 9.1.3 (ATLAS.ti Scientific Software Development GmbH, Berlin, Germany). We used a codebook approach to thematic analysis for the interview data.^23,24^ Two team members initially read through 6 transcripts to inductively develop an initial codebook. We collectively established initial codes and code definitions, iteratively revising them throughout the coding process. Three interviews were randomly selected and independently double-coded using the initial codebook. The team then met to discuss coding discrepancies. Four team members (AA, RL, MR, AR) built consensus around the codes, definitions, and application. The remaining interviews were randomly assigned and coded by 2 team members (RL or AR). The research team met regularly to debrief and discuss challenges until consensus was reached. After coding, the full team used an iterative, consensus-based approach to identifying themes and subthemes, collaboratively interpreting findings. We maintained trustworthiness through credibility (eg, debriefing with our Office of Superintendent of Public Instruction research partners), confirmability (eg, having multiple independent coders, consensus-based theme development), and audit trails (eg, maintaining detailed notes about coding, analysis, and decision points).^25^

## RESULTS

**Figure 1.**
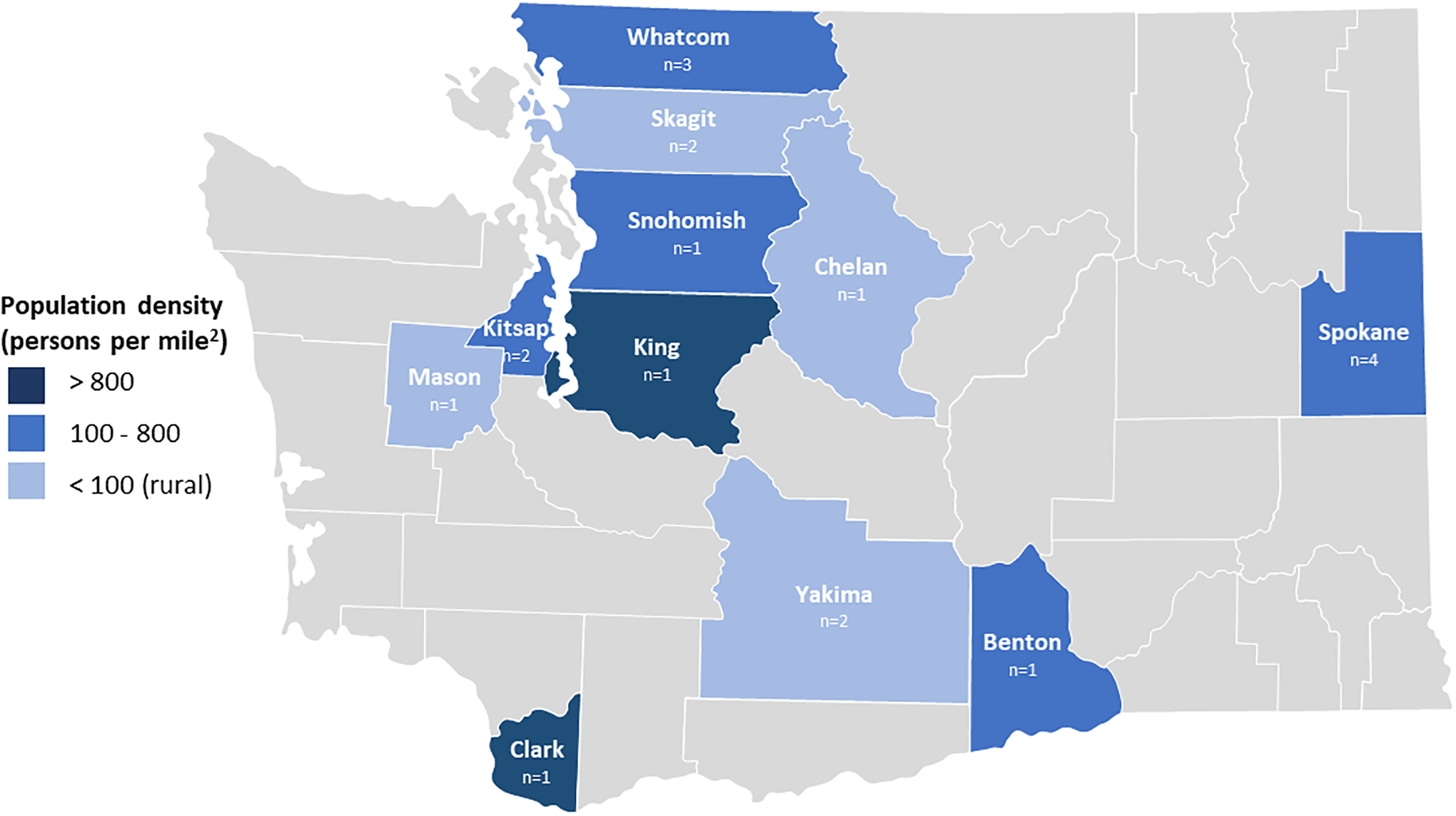
Participants by County and Population Density in Washington State Shaded counties indicate location of participants from ≥1 school district; lightest blue indicates rural county as defined by WA State law (ie, population density <100 persons per square mile or smaller than 225 square miles).

We interviewed 19 school leaders—7 superintendents and 12 school board members—representing 13 school districts across 11 counties of varying population density and rurality in Washington State (Figure 1). School districts ranged in size from 1694 to 23,067 students (Table 1). On average, 60% of the school districts’ students identified as White and 25% as Hispanic/Latino. Nearly half of students (47%) were eligible for free/reduced lunch, and 11% were English language learners.

We present results in line with our research questions to identify (1) concerns related to surveying students about SV and (2) potential solutions to mitigate concerns. While many participants expressed support for such survey efforts, our results focus on participant concerns to inform potential solutions for researchers and practitioners to conduct future work. In addition, while our research and interview questions focused specifically on SV surveys, participants often answered by discussing broader issues about SV programs/interventions and attitudes about topics related to SV (eg, gender identity), which is reflected in the quotes. For each category, we identified 3 themes with associated subthemes. Our analysis demonstrated no substantive differences in themes between participants from rural, suburban, and urban school districts, so results are presented together. Tables 2 and 3 provide additional illustrative quotes.

## CONCERNS RELATED TO SURVEYING STUDENTS ABOUT SV

### Theme 1: Community Norms and Misconceptions

#### Political or religious climate.

Participants described the political and/or religious climate that has led to certain topics, like SV, becoming more divisive. They reflected on how parents and families were scrutinizing school activities (eg, social-emotional learning) with a more critical eye, leading to a rise in pushback and criticism in the last few years alongside the COVID-19 pandemic and increased political polarization. Many participants mentioned it was a small—but vocal—minority of parents/caregivers opposed to SV research and prevention. Participants also noted a tendency for some community members to oppose anything perceived as liberal (including sexual health and violence prevention education). In the current climate, topics like sexual health and violence were being conflated with gender identity and critical race theory, generating pushback based on misconceptions of what was being discussed and a general distrust of the “agenda” of schools:

Given the political climate in the country right now, it doesn’t take much for community members to latch onto misinformation or to be rattled by something that they believe the school is doing … For example, a teacher was teaching critical thinking skills in her class and she abbreviated it to CTS, and she got quite a bit of pushback on that from some members of the community because they thought that the CTS, critical thinking skills, [was] similar to CRT, critical race theory.

(Superintendent)

For some communities, religious beliefs played an important role in attitudes around acceptability and appropriateness of surveying students about SV. There was a sense that for individuals holding strong religious beliefs, discussions about SV should happen within the home or within the extended religious family, so parents can choose how and when children engage in those discussions.

#### Tension about sexual health education.

School leaders described how the 2020 passage of comprehensive sexual health education legislation was an important context for discussing SV. The rollout of that education gave rise to resistance “mostly due to misunderstanding and misinformation about what that curriculum is” (school board member). Participants discussed how quickly rumors would spread through hearsay (eg, kindergarteners are being taught about sex), which would fuel reactionary backlash. Even if many districts were already teaching the same content about sexual health, the introduction of the new state standards ignited resistance.

### Theme 2: Parental/Caregiver Discomfort

#### Responsibility of parents versus schools.

Participants discussed the tension parents feel about their own responsibility versus the responsibility of schools in educating students about SV and related topics. There was a sense that parents/caregivers sometimes question why schools engage in certain topics, asking, “why is the school district in this lane?” (school board member) when there are survey questions related to attitudes about gender roles, for example. One school board member expressed how parents may get defensive or upset when they feel like their ability to have an important conversation with their child gets “pulled out from under them.”

#### Introducing students to SV too early.

Participants noted age was a primary concern for parents/caregivers, with middle school perceived as early for introducing topics like SV. There was concern that students may not be familiar with certain terminology (eg, “intercourse”) and school surveys should not be the initial exposure to these words. Parents were described as still viewing sixth-grade students “as not young adults, not teenagers, but still elementary age students,” especially at the beginning of the school year (school board member). However, many participants acknowledged middle school students do experience SV and recognized the importance of asking questions to understand their experiences. There was a tension some participants felt between feeling concerned a survey would expose students to topics youth don’t yet know and knowing that some students have already experienced forms of SV, particularly in the developmental period of middle school where students are at different phases and levels of exposure.

### Theme 3: Survey Language and Administration

#### Wording of survey questions as too explicit or reinforcing a gender binary.

Participants expressed concerns about specific language used in the survey questions, including phrases that were explicit or reinforced a gender binary. Concerns centered around descriptive definitions of sex (eg, “Sex includes oral (involving the mouth), vaginal (involving the vagina) or anal (involving the anus) sex”), which school leaders felt would generate negative interest from parents/caregivers who would find that inappropriate. Related to questions about normative gender roles and rape myths (eg, “a girl wearing revealing clothing deserves to have comments made about her”), some participants felt such language could reinforce binary and potentially harmful gender norms. One school board member said: “Guy/girl is simplified in a way that would be offensive to some members of our community.”

#### Traumatizing students and confidentiality issues.

Some participants expressed concerns about traumatizing students by asking sensitive questions about SV, especially for students who have experienced SV. One school board member noted the school may not know what the student or family is going through, so “that’s a very uncomfortable and inappropriate position to put that student in.” In addition, participants brought up the need to protect student data, especially around sensitive information, and to carefully consider what goes in health files and what might be public record.

## SUGGESTED STRATEGIES TO MITIGATE CONCERNS AND INCREASE ACCEPTABILITY

### Theme 1: Clear Messaging That Emphasizes Purpose, Safety, and Health

#### Providing clear motivation that builds trust.

Participants emphasized the need to provide clear motivation and purpose for surveying students about SV, as a means of building trust with the school community. Before launching a survey, education with parents/caregivers and students about why the survey is being done, who has access to the data, and how the information is being used is critical.

People need clear communication. I think sometimes when we put out surveys, I don’t think we always necessarily do such a good job of just making it simple and clear about why we’re doing it.

(Superintendent)

One school leader mentioned the idea that soliciting input from parents, for example, would be helpful to spread the word to other parents. Participants emphasized this communication should be done well in advance of survey administration to ensure parents/caregivers have time to understand what is happening and to ask questions. Some participants mentioned no matter how much education is done, there will still be some parents/caregivers who do not want their children to participate or will not find these efforts appropriate.

#### Reinforcing safety and health messaging.

Relatedly, participants underscored messaging around surveys should be about student safety or health, rather than emphasizing sexual terms. Participants felt safety was a “common core shared value and goal” for parents/caregivers (school board member). Participants also felt emphasizing healthy relationships or relationship violence would be more palatable compared to SV, given that the word “sexual” can raise concerns.

### Theme 2: Involving Parents and Students

#### Engaging school community (eg, parents, students) with data.

Participants recommended engaging parents and students with the data, so they can understand the importance of the survey and see what the survey results were used for. Some school leaders mentioned their districts are increasingly cognizant of making visible use of survey data once it is collected so there is a clear purpose for the survey. One school board member described how, for some students, seeing the use for survey results can be valuable:

What would really value [students’] time is to have the opportunity to see what a difference taking the survey meant, what you did with the results and whether you used it to advocate for certain things … to see what the results were and then what next steps you are taking.

#### Amplifying student perspectives that normalize SV discussions.

Given that students sometimes have differing opinions from administrators or parents/caregivers about SV prevention, school leaders emphasized that amplifying the student perspective may help build trust and provide a “countermeasure to parental angst” (superintendent). Having parents/caregivers hear directly from students that such surveys and conversations are useful and welcome may help to counter pushback.

The students want to talk about it, the students are embracing it, the students want to know how to help, what to look for, how to get help, how to have that conversation with a caring adult who can help facilitate and navigate through those awkward, horrible situations.

(School board member)

A couple of participants felt a balance was needed between elevating student voice and placing students in the middle of an argument to avoid inadvertently directing negativity at the students.

### Theme 3: Modifying Survey Language and Administration

#### Soften/tailor language.

Many participants recommended softening more explicit language in survey questions. For example, there were several questions where participants suggested removing the word ‘sexual’ (eg, “Made me do sexual things I didn’t want to”). Some participants saw these types of changes as tailoring questions to be more age appropriate for middle school students and palatable to community members.

#### Flexibility in survey administration.

In addition to changing question wording, participants recommended ensuring flexibility in survey administration, differentiating surveys for sixth-, seventh-, and eighth-grade students since eighth-grade students might have different vocabulary, maturity levels, and experiences than sixth-grade students. One school board member likened this to teaching math: “Obviously when we teach students about math, we don’t teach them the same thing in eighth grade as we do in sixth grade.” Timing during the school year was an important consideration. Participants recommended administering surveys in the spring versus the fall to give students time to acclimate to the school year, especially sixth-grade students who may be starting in a new school that year.

Definitely don’t ask these in the fall of any grade because the kids have all been home with their parents over summer. The parents, if anything, need the acclimation to school again, almost more than the kids do. Secondly, I’m not sure that you should ask these of sixth graders at all. Maybe the spring of seventh grade.

(School board member)

Ability to opt out for both parents and students was also seen as important. In addition to opting out of the survey entirely, having more nuanced opt-out options for certain questions or sections was recommended.

#### Alignment with existing school efforts.

Finally, participants emphasized surveys needed to align with existing school sexual health education. School leaders did not want survey questions to be the first introduction to certain words or ideas, so survey language needs to fit with what students have already learned in school (eg, by grade and by timing during the school year). Some participants brought up that teachers and staff would be more comfortable fielding questions and talking about the survey if topics like SV were already introduced in school. Surveys could be seen as valuable if they assessed student comprehension of the education.

## DISCUSSION

Our findings highlight concerns related to surveying students about SV from the perspective of superintendents and school board members and identify practical strategies to mitigate these concerns to support future success in SV surveys and evaluation. These real-world barriers associated with school-based SV research pose consistent challenges for researchers and practitioners.^26^ Furthermore, several participants commented on a recent rise in resistance and distrust amidst an increasingly polarized political climate and the COVID-19 pandemic. Strategies to facilitate SV research need to be responsive to current and changing context. Our results offer broad and contemporary considerations to inform strategies for researchers and practitioners when planning SV research and evaluation in middle schools.

Among the concerns that school leaders highlighted was parental/caregiver discomfort. Parents are vital to ensuring SV is taken seriously in schools, but parental engagement is often overlooked in school-based SV research and prevention.^18^ Our participants emphasized the need to engage parents by providing them clear purpose for surveying students, answering questions, and sharing back results from collected data. While school leaders are often the ultimate decision-makers about which surveys are implemented, some noted they had to be responsive to parental concerns, especially from vocal groups. This may mean altering or blocking efforts that could benefit students to appease parents. As participants suggested, focusing on student safety and health as underlying goals may help to allay some parental concerns. Participants reported parents often felt like it was their responsibility to address SV with their children rather than the responsibility of schools. While parents are an important source of information and model for youth attitudes/behaviors, parents may require specific resources and additional support to increase their confidence to have discussions on sensitive topics like SV.^27^ There can be tension between parents wanting to protect students from violence and wanting to protect their children’s innocence, leading to conflicting ideas of how to achieve “safety.”^17^

Participants noted age as a potential concern where middle school might be seen as too early to discuss SV. Accurate and developmentally appropriate education on sexuality and healthy relationships helps to promote healthy sexual development for youth and provides a foundation for improved sexual health into adulthood.^28^ Current sexual health education curricula are often inadequate, and youth feel ill-equipped to pursue healthy romantic relationships.^29^ In addition, studies on the timing of adolescent-parent discussions have identified middle school as an ideal time to discuss sexuality topics (eg, dating relationships, safe sex, sexting, consent, SV).^11,16,17,30^ To mitigate concerns around age, participants recommended differentiating surveys for sixth-, seventh-, and eighth-grade students, which can then be tailored to the vocabulary and maturity levels of students in those grades. Given that patterns of violence change across middle school years, with increasing victimization from 6th through 8th grades,^31^ this approach may be effective, even though students may be at varying developmental stages in each grade.

Participants raised concerns about potentially traumatizing students, especially those with a trauma history, by asking sensitive questions about experiences of violence. Research shows relatively low prevalence of distress among adolescents from taking surveys on sensitive topics like violence and maltreatment, with distress stemming from concerns about confidentiality/privacy, feeling awkward or weird, or being reminded of something.^32–34^ Generally, research on violence is well-tolerated by adolescents, who are capable of assessing potential risks for themselves and often find research to be positive, enjoyable, reflective, and even cathartic.^32,35,36^ Participants’ suggestions to mitigate potential negative reactions—for example, to involve and amplify students’ perspectives and to have flexible opt- out options—align with recommendations from prior research.^32,36,37^ Indeed, having youth involved in or leading SV survey efforts may help to counter resistance, increasing acceptability and sustainability of SV research and prevention.^38^

### Implications for school health policy, practice, and equity

Our findings have important implications for rigorous evaluation of school SV prevention programs. Discouraging surveying of students early in the school year makes it difficult to conduct pre-and post-surveys to assess program impact. This may necessitate restricting intervention programming to the second half of the school year or using other study designs for SV program evaluation. Concerns about SV surveys may mean research is conducted among increasingly selected schools and populations willing to engage with sensitive topics, limiting external validity of evaluation findings. Schools may also be unable to gauge prevalence of SV among students, rendering this already hard to identify public health problem more invisible and limiting potential resource allocation. Given the level of concern voiced by some leaders about conducting SV surveys in middle schools, there may be opportunities for schools to leverage surveys on broader topics, such as school climate or bullying, that may be seen as more acceptable and still able to support policies and practices that ultimately contribute to student safety and SV prevention.^39–43^ Outside of conducting surveys to establish the need for SV education, national health and sexuality education standards can help to promote the implementation of SV programs that are aligned with adolescent development.^12,13^

### Limitations

While we attempted to capture perspectives of school district leaders from diverse geographic regions with purposive sampling, our study was conducted only in Washington State. Given the importance of political, religious, and cultural norms in attitudes about SV, results may differ across populations in other states. However, participants across school districts shared common concerns, regardless of geographic region. Participation in the study was based on willingness to be interviewed, so our sample was generally in favor of SV efforts in schools. We may not have captured perspectives of school district leaders most opposed to SV surveys. While we reached sufficient information power to answer our research questions, future research should consider how to further diversify the sample population and assess concerns in districts representing more divergent views.

### Conclusions

Our study identifies concerns about surveying students about SV and highlights avenues for researchers and practitioners to more effectively conduct school-based SV research, particularly in middle schools. Engaging and educating all school community members (eg, school leadership, school boards, school staff, parents, students) to build trust, in addition to carefully considering survey language and administration, are important steps to increase acceptability of such efforts. Our results also point to the importance of understanding and attending to political and cultural norms within communities, including the spread of misinformation, as they relate to SV. These data can help researchers and schools anticipate and navigate concerns school community members might have in implementing SV surveys for students.

## Supplementary Material

Supplement **Data S1.** Supplementary Information.

## Figures and Tables

**Table 1.** Characteristics of school districts from which participants were drawn

	All school districts (n=13)	
	Mean (Range)	
**Student gender**		
Male	51.4%	(48.8% - 53.0%)
Female	47.7%	(43.2% - 49.1%)
Gender X	0.8%	(0.0% - 8.0%)
**Student race/ethnicity**		
White	60.3%	(37.1% - 81.4%)
Hispanic/Latino	24.9%	(5.6% - 56.3%)
Two or more races	6.7%	(2.2% - 12.3%)
Asian or Asian Pacific Islander	4.4%	(0.3% - 24.6%)
American Indian/Alaska Native	1.2%	(0.2% - 5.1%)
Black or African American	2.0%	(0.4% - 8.8%)
Native Hawaiian or other Pacific Islander	0.6%	(0.0% - 2.8%)
**Number of students**	9,136	(1,694 – 23,067)
**Student-teacher ratio**	16:1	(13:1 – 18:1)
**Student eligible for free/reduced lunch**	47.1%	(9.1% - 69.2%)
**English language learners**	10.9%	(1.1% - 24.6%)

Source: Washington Office of Superintendent of Public Instruction data portal for the 2021–2022 school year

**Table 2.** Illustrative quotes of concerns related to surveying middle school students about sexual violence (SV)

Theme	Subtheme	Illustrative quotes
**Community norms & misconceptions**	Political or religious climate	“I feel like there’s a big shift where there’s not a lot of trust, …parents are not trusting the systems … I don’t even know what caused that, but … there’s a big disconnect right now between some parents and the education system, and I think there always has been to some degree, but with the polarization of everything it’s become more intense.” [School board member] “[The concern or the pushback is] going to come from our very religious and very conservative… they’re two different groups. There’s an overlap obviously. But our very religious group is not necessarily our very, very vocal anti-CRT [critical race theory], anti-SEL [social-emotional learning], … anti-everything. Our religious group tends to be more specifically focused on concerns and appropriateness than our ultra conservative group.” [School board member]
	Tension about sexual health education	“[Comprehensive sexual health education] got passed a couple years ago, and that is the major pushback that we are experiencing at the local and state level. Mostly due to misunderstanding and misinformation about what that curriculum is. There’s a bit of an environment, particularly in conservative faith communities, that this is introducing things or teaching kids how to have sex way too early or teaching kids, or indoctrinating kids into LGBTQIA lifestyles or identities.” [School board member] “I think what’ll happen is somebody’s at some community event and starts talking about, “Can you believe what they’re teaching kids about sex ed” and somebody else says, ‘Yeah, I opted my kid out’ and they said, ‘Well, I’m going to opt my kid out too.’… And the reality is … the new state curriculum around sex ed, most districts have been teaching that for years and there wasn’t any concern about that. And now all of a sudden everybody’s like, ‘Oh my gosh.’” [Superintendent]
**Parental/ caregiver discomfort**	Responsibility of parents vs. schools	“We are asking school districts in general to take on a lot around culture, around sex, around various norms in our communities. The more questions that there are like this, the more I could imagine some parents saying, “Why is the school district in this lane?” We get that already for different things, but because there’s so much here … I could imagine parents and community members saying, “Why is the school district asking questions about household chores and the role of men with women and children?” [School board member] “I think parents start to become defensive and upset when they feel like their ability to lead this important conversation got pulled out from under them. It’s being discussed as parental rights, but it’s more about, “I’m entrusted with this really important thing and I’m caring and raising these humans and they’re amazing and beautiful and perfect and I want to like keep that going.” [School board member]
	Introducing students to SV too early	“At the middle school level, [the parents] are highly, highly protective. And in our community, I think they see 11–15 year olds as being very vulnerable and very young. Talking about these kinds of issues, we are scaring them. We’re talking to them about things that they don’t need to know about yet.” [School board member] “At sixth grade the parents would still view their children as not young adults, not teenagers, [but] still elementary age students. In some school districts, you’re also going to find that sixth graders are still in an elementary environment, not in a middle school. That has a lot to do with the perception of the students. But for… those seventh and eighth grade students who are being more outwardly observable in relationships, then I think this would be an appropriate question.” [Superintendent]
**Survey language & administration**	Wording of survey questions as too explicit or reinforcing a gender binary	“When you start getting into the different types of sexual activities [that] may involve some concern, more descriptive, and then obviously the anal sex, that would prompt some [negative] interest from groups of community and parents.” [Superintendent] “The guy/girl is simplified in a way that would be offensive to some members of our community. And even I think the board would have issues with this. This would not be consistent with our values in our district improvement plan that is very much focused on equity because not all students would see themselves in these questions.” [School board member]
	Traumatizing students and confidentiality issues	“If we put questions like this in front of a child who has been abused sexually already and is already dealing with that personally and in their family and maybe beyond what we might know about as a school, that’s a very uncomfortable and inappropriate position to put that student in.” [School board member] “Anything that’s public, we want to make sure that it’s held in a way that it doesn’t pinpoint or share information that could potentially showcase that particular student or population group.” [Superintendent]

**Table 3.** Illustrative quotes of strategies to mitigate concerns and increase acceptability of surveying middle school students about sexual violence (SV)

Theme	Subtheme	Illustrative quotes
**Clear messaging that emphasizes purpose, safety, and health**	Providing clear motivation that builds trust	“People need clear communication. Sometimes when we put out surveys, I don’t think we always necessarily do such a good job of just making it simple and clear about why we’re doing it, what we’re doing with the information, who has access to it, or does not have access to it, and how that directly is going to come back on their kid. The [parents] that do have those concerns will want to know that information.” [Superintendent] “Soliciting stakeholder input, I think that will help where you have a focus group with diverse parent representation, just having that conversation, because they’ll talk to each other and to other people, they’ll talk to friends. If you have a key communicators group that really understands the point of the survey and why collecting this kind of data is actually helpful and that it is not about sexualizing anything at the middle school. This is not about pushing a particular agenda; this is not about forcing students to embrace a particular set of values related to gender diversity. This really is about protecting students, making sure that they’re safe.” [Superintendent]
	Reinforcing safety and health messaging	“I am not familiar with any parent who doesn’t want their kid to be safe. That’s a common core shared value and goal …I would encourage reflecting, ‘Here’s what we are hearing from students. Here’s what we understand from the…surveys we do. Here’s what we know from our own population and this is important to us because your kids being safe is important to us.’ One of the ways that we can help them be more safe is having a real picture of what they’re experiencing and that’s what this survey is going to help us understand.” [School board member] “From the standpoint of being able to educate our families at the middle school, it’s about relationship violence prevention. I think the word sexual can be taken off and just talk about what a healthy relationship is as we complete these surveys. Because this tells me more than just sexual violence, right? This tells me about somebody that is treated unfairly or is bullied or made to do something they were not comfortable doing. Which goes back to the health and development of what is consent? What does a healthy relationship look like?” [Superintendent]
**Involving students & parents**	Engaging school community (e.g., parents, students) with data	“We’ve become really involved in the use of data, the whole idea of individualizing education for students. We look at individual students in every piece of data that we can glean and then try to build systems to support individual students in groups of students. We use survey data to either support students or build systems that are going to support large groups of students. There was a time that we would just survey to survey, but we wouldn’t do anything with the data. I think public education has gotten by [with] that. Now if we’re going to survey, then there’s almost an unwritten rule or expectation that we are going to evaluate that data.” [Superintendent]
	Amplifying student perspectives that normalize SV discussions	“I think that’s a great countermeasure to parental angst when we do intentionally include student voice. Our kids learn all sorts of stuff outside the walls of our schoolhouses, at dinner table with parents. They’re learning all sorts of stuff - good, bad, ugly, whatever…that’s eye opening for parents when they realize that. Frankly, many of our students are very good at just saying, this is okay and this is normalized to be able to have these kinds of discussions with their peers.” [Superintendent] “We want to elevate student voice, but we also know that it’s a pretty nasty world out there. We’re always mindful, how do we elevate student voice without putting our students inadvertently into becoming targets or in the crosshairs that will last for them a lifetime, potentially? That’s where parents and students have to think through on a family by family basis.” [School board member]
**Modifying survey language & administration**	Soften/tailor language	“If this is sixth through eighth [grade students], I would probably reword the “made me do sexual things” and “showed friends and posted pictures naked or doing something sexual.” I think it could be more age appropriate with the same understanding and meaning that didn’t maybe come off as quite as harshly received by our community.” [School board member] [Instead of “Made me do sexual things I didn’t want to”] How about just, “Made me do things I didn’t want to. Or made me do things I was uncomfortable doing.” [School board member]
	Flexibility in survey administration	“I think definitely these would be more eighth grade type of questions. And, it does matter when, because in the fall, especially for sixth graders, they’re coming into a new school most of the time. So, there’s not a lot of trust necessarily with the staff yet. It hasn’t had a chance to be established. Whereas an eighth grade cohort, in theory, many of those kids have maybe been in that school for a while. If it’s the counselor that’s helping facilitate this, maybe it’s somebody that they trust and maybe there’s some rapport with that person and the families too around that.” [Superintendent] “I wonder if there’s even the ability to say like, ‘Fine for my kid to take this part of the survey, but not this part of the survey’…that full binary opt-in, opt-out can really exclude an important data set, especially if it’s a more conservative family, or for whatever reason, the family hasn’t yet had this conversation within the family.” [School board member]
	Alignment with existing school efforts	“I would not want any of these questions going out in my school district without it aligning with our other curriculum…I don’t want to be the first time they are asked these kinds of questions. A prerequisite to any of these questions has to be in line with what’s being taught already in the school with the state required bill that was passed regarding sex ed education.” [School board member] “I’m not sure what they talk about in sex ed in sixth and seventh grade. So if these topics have already been talked about and it’s been introduced with teachers who know how to talk about this stuff, then asking these questions I think really is valuable to us. Did we get through to the kids or not? It comes in a context as opposed to a series of questions.” [School board member]
