# Supplementary material for: School District Leader Perspectives on Surveying Middle School Youth About Sexual Violence
Source: J Sch Health. Author manuscript; Available in PMC 2025 Jan 18. (PMC11739540; doi:10.1111/josh.13496)
Supplement: Supplement — Data S1. Supplementary Information. [file NIHMS2045615-supplement-Supplement.docx]

**Supplement**. Interview guide with example survey questions shown to participants in interviews

First, we’d like to ask you about your role at your school or within the district. What is your role?

Next, we’d like to ask about how sexual violence prevention efforts are implemented.

- Who makes decisions about what sexual violence prevention efforts and surveys are implemented in your district? At the school level?
  - How might this differ in middle schools compared with high schools?
  - Is this formal policy or informally done?
- How have you been involved, if at all, in decisions about which programs and survey are done in the district or school?
- What do you think the school community thinks about surveying students about SV?
  - How might this differ in middle schools compared with high schools?
  - How might attitudes differ between school staff and caregivers or the wider community?

We sent our survey questions to you to give you a better sense of the wording and language that is being used to collect data on experiences of violence. These have been adapted from other surveys in the country that have been given to middle school students.

I’m going to share my screen and show you a few specific questions to get your thoughts:

In the past 12 months, how often has someone you were in a dating relationship with done any of the following?

In the past 12 months, how often has someone you were in a dating relationship with done any of the following? *(Miller et al., 2020; Straus et al., 1996)*

|  | Never | A few times  (1-3 times) | Sometimes  (4-9 times) | Often  (10+ times) |
| --- | --- | --- | --- | --- |
| Called me hurtful names, like ugly or stupid |  |  |  |  |
| Told me not to talk to others or told me who I could hang out with |  |  |  |  |
| Physically hurt me (like shoving, grabbing, slapping, punching, choking) |  |  |  |  |
| Threatened to hurt me if I didn't do what they wanted me to do |  |  |  |  |
| Made me do sexual things I didn’t want to (like kissing, touching, oral sex, intercourse) |  |  |  |  |
| Yelled at me or intentionally destroyed something that belonged to me |  |  |  |  |
| Showed friends or posted pictures of me naked or doing something sexual |  |  |  |  |

Have you ever seen someone about your age pressure someone else to kiss, touch, or have sex when they did not want to? Sex includes oral (involving the mouth), vaginal (involving the vagina) or anal (involving the anus) sex. *(Healthy Youth Survey: Form B, 2021)*

In the past 12 months, how often has anyone you know done the following to you IN PERSON? *(American Association of University Women, 2011; Miller et al., 2020)*

|  | Never | A few times  (1-3 times) | Sometimes  (4-9 times) | Often  (10+ times) |
| --- | --- | --- | --- | --- |
| Made unwelcome or unwanted sexual comments, jokes, gestures, or looks |  |  |  |  |
| Called you gay or lesbian in a negative way |  |  |  |  |
| Touched you in an unwelcome or unwanted sexual way |  |  |  |  |
| Showed, gave, or left sexual pictures, drawings, messages, or notes that you didn’t want to see |  |  |  |  |
| Spread sexual rumors about you, whether or not they were true (like telling others that you have done sexual things) |  |  |  |  |
| Forced you to do something sexual (like kissing, sexual touch, oral sex, or intercourse) |  |  |  |  |

| 1  Strongly disagree | 2  Disagree | 3  Agree | 4  Strongly agree |
| --- | --- | --- | --- |

How much do you agree with the following statements (*Adapted from the Illinois Rape Myth Acceptance Scale (Payne, Lonsway, & Fitzgerald, 1999; Cook-Craig et al., 2014)*:

- Girls should have sex with the guy they are dating when he wants
- If a guy spends money on a date, the girl should have sex with him
- Guys should respond to challenges to authority by a date or girlfriend by insulting them or putting them down
- If a girl if sexually assaulted while drunk, she is to blame
- Sexual assault charges are often used as way of getting back at guys
- Many girls lead a guy on, and then claim it was sexual assault
- When girls are sexually assaulted, it is often because the “no” was unclear

| 1  Strongly disagree | 2  Disagree | 3  Agree | 4  Strongly agree |
| --- | --- | --- | --- |

How much do you agree with the following statements (*Gender equitable attitudes scale (Hill et al., 2022 based on prior measures from McCauley & Miller, 2014; Pulerwitz & Barker, 2008; Chu, Porche, & Tolman, 2005)*:

- A guy takes responsibility for his actions.
- A guy never needs to hit another guy to get respect.
- A girl wearing revealing clothing deserves to have comments made about her.
- It bothers me when a guy acts like a girl.
- Guys should sleep with as many girls as possible.
- If a guy tells people his worries, he will look weak.
- In a good dating relationship, the guy gets his way most of the time.
- Guys should only have sex with girls.
- I can respect a guy who backs down from a fight.
- I would be friends with a guy who is gay.
- A guy should share in household chores (for example washing dishes, vacuuming).
- If a girl is raped (forced to have sex) it is often because she did not say "no" clearly enough.
- Guys put women and children first.
- What concerns, if any, do you have about students answering survey questions related to violence? About unwanted sexual activity?
  - Are there particular words or language that you have concerns about?
- What concerns, if any, do you have about these questions?
  - Do you have any concerns specifically for younger students or those in lower grade levels, such as 6^th^ grade?
- What types of survey questions might provide useful information to the school and district?
  - Would these questions be acceptable to ask students? Parents? School board members?

Thank you for sharing the concerns you might have about surveying students about sexual violence. We want to ask now about what we can do as a research team to collect this important data while being respectful of the school community.

- Do you have any thoughts about how to lessen concerns about surveying students about sexual violence?
  - Are there wording changes you would suggest to the survey questions above?
  - How do you think students can be involved to help address these concerns?
    - For example, would having data from students saying the questions were comfortable for them to answer alleviate concerns?
- How can we communicate the value of asking these questions to parents and the school community?
  - What kind of language might you use if talking to students or families about sexual violence prevention?
  - How would you word or frame an invitation to participate in surveys about sexual violence to students/families?
